# Biocompatible Copolymerized Gold Nanoclusters: Anti-*TNF-α* siRNA Binding, Cellular Uptake, Cytotoxicity, Oxidative Stress and Cell Cycle Effects In Vitro

**DOI:** 10.3390/biomimetics10120812

**Published:** 2025-12-04

**Authors:** Jananee Padayachee, Moganavelli Singh

**Affiliations:** Nano-Gene and Drug Delivery Group, Discipline of Biochemistry, University of KwaZulu-Natal, Private Bag X54001, Durban 4000, South Africa; padayacheej1@ukzn.ac.za

**Keywords:** siRNA, gold nanoclusters, triple-negative breast cancer, cytotoxicity, gene therapy, cellular uptake, TNF-*α*

## Abstract

Small interfering RNAs (siRNAs) have emerged as a powerful tool in the treatment of aggressive cancers. By exploiting and mimicking the natural gene regulation mechanism of RNA interference (RNAi), they allow for sequence-specific silencing of aberrant genes. siRNA-mediated knockdown of the inflammatory cytokine tumour necrosis factor-alpha (TNF-α) presents a novel therapy for triple-negative breast cancer (TNBC). This study investigated the potential of novel biomimetic glutathione-synthesised gold nanoclusters (AuNCs) as siRNA delivery vehicles. AuNCs were functionalized with biocompatible chitosan and polyethene glycol, and their interactions with siRNAs were investigated through binding studies. In vitro cytotoxicity and cellular uptake were conducted in the human breast cancer (MCF-7), TNBC (MDA-MB-231), and embryonic kidney (HEK293) cells, while the effect of anti-*TNF-α* siRNA nanocomplexes on biological processes, such as oxidative stress, apoptosis, and cell cycle distribution, was investigated using flow cytometry. UV–visible and Fourier transform infrared spectroscopy, as well as transmission electron microscopy, confirmed the synthesis and functionalization of the AuNCs. Functionalized AuNCs (FAuNC) effectively bound and condensed siRNA and protected against nuclease degradation. AuNCs facilitated efficient cellular uptake and were well-tolerated in vitro. Anti-*TNF-α* siRNA treatment of the MDA-MB-231 cells increased apoptosis and oxidative stress levels, and affected cell cycle distribution. Although the overall knockdown was low, these FAuNCs exhibited favorable physicochemical characteristics, low cytotoxicity and good cellular uptake in vitro, warranting further optimisation for improved delivery of therapeutic siRNAs.

## 1. Introduction

Small interfering RNAs (siRNAs) have been widely studied as triggers of RNA interference (RNAi), a sequence-specific gene silencing process in eukaryotes that regulates gene expression at the post-transcriptional level and protects against infections by viruses and transposable elements [1]. These siRNAs are short (21–22 nucleotide) molecules with 3′ overhangs, formed through cleavage of larger dsRNA molecules. Following cleavage, they are incorporated into a multiprotein complex called the RNA-induced silencing complex (RISC). The guide strand of the siRNA directs the RISC to cleave its complementary mRNA, thus knocking down gene expression [2]. The ability of siRNA-mediated RNAi to silence the expression of any gene of interest has led to its widespread application in various fields, including functional genomics studies, pest control, and therapeutic targeting of disease-causing genes [1].

For therapeutic use, siRNAs can be designed to bear complementarity to the mRNA of any disease gene. They are chemically synthesised to mimic the structure and length of natural siRNAs produced after cleavage, or the larger dsRNA molecules, which are substrates for cleavage [3]. This enables the efficient incorporation of exogenous siRNAs into the cellular RNAi machinery, resulting in potent, sequence-specific gene knockdown.

siRNA therapies show particular promise in treating diseases previously considered undruggable and cancers with high mutagenic diversity, such as triple-negative breast cancer (TNBC). However, the in vivo knockdown efficacy of siRNA is limited by the unfavourable physicochemical properties of siRNA molecules. Naked siRNA is highly susceptible to nuclease degradation and removal from circulation, and the large size and anionic charge of the molecule inhibit cellular uptake [4]. Much research has focused on developing suitable carriers that can protect siRNA and deliver it to the cytoplasm of target cells to mediate gene silencing.

Inorganic metallic nanoparticles (NPs), particularly those synthesized from gold (Au), have been widely studied as delivery vehicles for oncotherapeutics. Gold nanoclusters (AuNCs) are novel, ultrasmall NPs composed of a few to hundreds of atoms. This small size imparts unique characteristics to them compared to conventional NPs. Notably, AuNCs display unique optical properties, such as strong photoluminescence, that facilitate their use as imaging agents [5]. Compared to conventional fluorophores such as dyes and quantum dots containing heavy metals, AuNCs display good photostability, resistance to photobleaching, low toxicity, tunable emission wavelengths, and a large Stokes shift, reducing crosstalk between the emission and excitation wavelengths [6,7,8]. Recent studies have also demonstrated the potential of AuNCs as vehicles for gene therapy delivery. AuNCs capped with the positive oligopeptides have been shown to be capable of assembling with DNA and RNA [9], and of delivering siRNA for the knockdown of nerve growth factors in pancreatic cancer [10]. Plasmid DNA delivery has also been achieved using PEI-functionalized AuNCs [11] and PEI functionalized with sulfhydryl groups (PEI-SH) to allow for binding to the AuNC [12].

The tumour necrosis factor alpha (TNF-α) is a pleiotropic cytokine with a central role in the inflammation process [13]. While originally defined according to its role as an anticancer agent, it has since been shown to display both anti- and pro-tumour effects depending on the context and physiological concentration [14,15]. Low TNF-α levels present in the inflammatory tumour microenvironment have been implicated in promoting the development, growth, and spread of several cancers, including TNBC [14,16]. TNF-α has been shown to contribute to the aggressiveness of TNBC by activating growth-promoting pathways that induce invasion and metastasis, and the generation of cancer stem cells that promote relapse [17]. The cytokine thus presents a novel target for treating TNBC.

This study aimed to investigate the potential of AuNCs as siRNA delivery vehicles. Glutathione (GSH) reduction of HAuCl_4_ enabled the synthesis of ultrasmall, highly fluorescent AuNCs. GSH is considered a biomimetic peptide, having antioxidant properties. It is a tripeptide containing glutamate, cysteine, and glycine, which are essential for cellular metabolism. GSH intracellular concentrations can reach extreme levels in cancer cells. The GSH redox cycling enzymes play a crucial role in detoxifying cellular free radicals and non-radical species [18]. The AuNCs were further coated with the biocompatible and biodegradable polymer chitosan (CS) and the steric stabilizer polyethene glycol (PEG) at two weight ratios. CS-based biomimetic nanocomposites are being utilised in wound healing, hemostasis, and therapeutic delivery due to their ability to facilitate adhesion, release, and adsorption [19]. CS is a natural cationic polymer commonly used in gene delivery due to its biodegradability, biocompatibility, and strong interactions with nucleic acids, which are facilitated by the presence of positively charged amino groups [20]. Addition of the stealth polymer PEG may lead to reduced opsonisation and thus increased circulation times in vivo [21]. Although PEG is synthetic, it can be designed to mimic biological functions in vivo, influencing its biomimetic applications. The ability to be modified with therapeutic biomolecules can enable its use in biological scaffolds such as hydrogels, drug delivery systems, and tissue engineering [22]. Additionally, it has no potentially toxic functionalities and possesses hydroxyl groups at the end of the chain, allowing for the addition of other biomolecules [23]. These functionalized AuNCs (FAuNCs) were further evaluated in vitro for cytotoxicity, cellular uptake, oxidative stress, and cell cycle analysis in the non-cancer HEK293, hormone receptor-positive breast cancer MCF-7, and TNBC MDA-MB-231 cells. This study provided a proof-of-principle demonstration of the potential of AuNCs in therapeutic siRNA delivery to breast cancer cells.

## 2. Materials and Methods

### 2.1. Materials

Gold (III) chloride trihydrate (HAuCl_4_·3H_2_O), L-glutathione reduced, CS (medium MW, 75–85% deacetylated), Bicinchoninic acid (BCA) kit and 14 kDa dialysis tubing were obtained from Sigma Aldrich (St Louis, MO, USA). Polyethylene glycol 2000 (PEG_2000_), Tris-hydroxymethyl-aminomethane (Tris-base), sodium dihydrogen phosphate (NaH_2_PO_4_), 4-(2-hydroxyethyl)-1-piperazineethanesulfonic acid (HEPES), ethylenediaminetetraacetic acid (EDTA disodium salt), sodium dodecyl sulphate (SDS), bromophenol blue, ethidium bromide, sucrose, dimethylsulfoxide (DMSO) and 3-[4,5-dimethylthiazol-2-yl]-2,5-diphenyltetrazolium bromide (MTT) reagent were purchased from Merck (Darmstadt, Germany). Ultrapure-grade agarose was purchased from Bio-Rad (Hercules, CA, USA). Anhydrous glycerol was obtained from Sisco Research Laboratories Pvt. Ltd. (Maharashtra, India). RNase A was obtained from Calbiochem (San Diego, CA, USA). The BLOCK-iT™ fluorescent Oligo and Lipofectamine™ 3000 (LF3K) were purchased from Invitrogen, Thermo Fisher Scientific (Waltham, MA, USA).

Minimum Essential Medium (MEM) was obtained from Gibco, Thermo Fisher Scientific (Waltham, MA, USA). Dulbecco’s Phosphate-Buffered Saline (PBS) was purchased from Capricorn Scientific GmbH (Ebsdorfergrund, Germany). Foetal Bovine Serum (FBS) was purchased from Cytiva (Marlborough, MA, USA). Trypsin-versene and penicillin-streptomycin were obtained from Lonza BioWhittaker (Walkersville, MD, USA). The Muse^®^ Caspase 3/7, Oxidative Stress, and Cell Cycle kits were purchased from Luminex Corporation (Austin, TX, USA). All sterile cell culture plasticware was purchased from Nest Biotechnologies (Wuxi, China) and Corning Incorporated (New York, NY, USA). The human embryonic kidney (HEK293), breast adenocarcinoma (MCF-7), and triple-negative breast cancer (MDA-MB-231) cell lines were originally obtained from the American Type Culture Collection (Manassas, VA, USA) and were tested for mycoplasma prior to in vitro studies. The 5× siRNA buffer, nuclease-free water, siGENOME non-targeting siRNA #1, and the ON-TARGET plus Human TNF (7124) SMARTpool siRNA were purchased from Dharmacon (Lafayette, CA, USA). The sequences of the individual siRNA duplexes are shown in Table 1. All other reagents were of analytical grade, with ultrapure 18 Mohm water being used in all experiments.

### 2.2. Synthesis, Functionalisation of AuNCs and Nanocomplex Formulation

#### 2.2.1. Synthesis of AuNCs

AuNCs were synthesised according to that reported in the literature [24]. Briefly, 20 μM HAuCl4 (1 mL) was mixed with 300 μL of a 100 μM GSH solution and 8.7 mL of deionised water under gentle stirring at room temperature. The solution attained an orange colour upon the addition of GSH, becoming colourless with stirring. The solution was maintained at 70 °C with stirring in a glycerol bath for 24 h. The resulting AuNCs were purified by centrifugation at 16,000× *g* for 1 min, to remove any large NPs, and then dialysed against deionised water (14 kDa MWCO) over 48 h, to remove unreacted gold ions and GSH molecules.

#### 2.2.2. Functionalisation of AuNC

AuNCs were functionalised with CS (AuCS) and PEG using two weight ratios (1% and 2% *w*/*w*). Functionalization with CS (75–85% deacetylated) was carried out according to the literature [25]. Briefly, a CS solution (2 mg/mL in 1% acetic acid) was added dropwise to 2 mL of the AuNCs with stirring, to a final concentration of 1 mg/mL. The solution was then diluted to 5 mL with deionised water, adjusted to pH 6.5 using 1 M NaOH, and stirred at room temperature for 30 min. The resulting AuCS NPs were dialysed as described previously.

Functionalisation with PEG was carried out using PEG conjugated to 1,1-carbonyldiimidazole (PEG-CDI). The appropriate volume of a 1 mg/mL stock PEG-CDI solution was added to 1 mL of CS (2 mg/mL) and stirred overnight to allow for the binding of the PEG-CDI to the CS. The resulting CS-1% PEG and CS-2% PEG solutions were then used to produce AuCS-1% PEG and AuCS-2% PEG following the protocol as for AuCS synthesis.

#### 2.2.3. Preparation of Nanocomplexes

Nanocomplexes were prepared by mixing varying amounts of the FAuNCs with a constant amount of siRNA (0.30 μg). Solutions were prepared to a final volume of 10 μL with HEPES-buffered saline (HBS) (20 mM HEPES, 150 mM NaCl, pH 7.4) and incubated at room temperature for 1 h to allow for binding. These nanocomplexes were used in the ensuing assays.

### 2.3. Characterisation of Nanoclusters and Nanocomplexes

The absorbance spectra of the AuNCs and FAuNCs were analysed by UV–visible (UV-vis) spectroscopy using a Jasco V-730 Bio Spectrophotometer (JASCO Corporation, Hachioji, Japan). Transmission electron microscopy (TEM) was used to determine the size and morphology of AuNCs and FAuNCs. Viewing was conducted on a JEOL T1010 TEM (JEOL, Tokyo, Japan), using samples that were air-dried on a 400-mesh copper grid. ImageJ 1.54q software was used to analyse the images and determine the average diameter of the NPs. Fourier-transform infrared spectroscopy (FTIR) was conducted using a Perkin Elmer Spectrum 100 FT-IR spectrometer (PerkinElmer Inc., Waltham, MA, USA) fitted with a universal ATR sampling accessory. The peaks in the spectra and the corresponding bonds were identified through comparison with the literature. The hydrodynamic diameters and zeta potentials of the NPs and their nanocomplexes were determined by nanoparticle tracking analysis (NTA) in a Nanosight NS500 (Malvern Instruments, Worcestershire, UK), using samples diluted (1:1000) in deionised water. The polydispersity indices (PDI) were calculated using Formula (1).

(1) $$PDI={\left(\frac{SD}{Mean}\right)}^{2}$$

### 2.4. Binding Studies

#### 2.4.1. Band Shift Assay

The assay was conducted as described in the literature [26]. Nanocomplexes were prepared as described in Section 2.2.3. After incubation, 2 μL of a gel loading dye (40% sucrose, 0.25% bromophenol blue) was added to the nanocomplexes. The nanocomplexes were then run in 1× Tris-borate-EDTA (TBE) buffer using a 2% agarose gel containing 2 μL of ethidium bromide (EB) (10 mg/mL) at 50 V for 30 min. Gels were viewed under UV light (λ = 300 nm) using a Vacutec Syngene G: Box BioImaging system (Syngene, Cambridge, UK), and images were captured with GeneSnap software, version 7.05.02 (Syngene, Cambridge, UK). The optimum, sub-optimum, and supra-optimum siRNA:FAuNC (*w*/*w*) ratios were determined and studied in nuclease protection and in vitro assays.

#### 2.4.2. Ethidium Bromide (EB) Intercalation Assay

Dye displacement studies [26] were conducted using the Glomax^®^-Multi Detection System (Promega Biosystems, Sunnyvale, CA, USA). Briefly, 100 μL of HBS was added to a 96-well flat-bottom black FluorTrac plate. Approximately 2 μL of EB (100 μg/mL) was added to the HBS, and the fluorescence was measured at excitation and emission wavelengths of 525 nm and 600 nm, respectively, to establish a baseline. Thereafter, 0.3 μg of siRNA was added to the wells and mixed to allow for binding. The fluorescence was measured to establish the maximum fluorescent intensity of the EB-bound siRNA and was set at 100%. The FAuNCs were then added to the solution in 1 μL increments, and fluorescence was recorded after each addition until a plateau was reached. The relative fluorescence was then calculated using Formula (2).

(2) $$F_{R}\left(\%\right)=\left(\frac{F_{i}-F_{0}}{F_{max}-F_{0}}\right)\times 100$$

where F_0_ = baseline fluorescence; F_i_ = fluorescent intensity following addition of FauNC; and F_max_ = maximum fluorescent intensity of intercalated siRNA. The approximate siRNA:FAuNC weight ratio was determined after each addition, and the data were plotted as the relative fluorescence against the siRNA:FAuNC ratio.

#### 2.4.3. Nuclease Protection Assay

Nanocomplexes were prepared (Section 2.2.3) at the optimum, sub-optimum, and supra-optimum ratios obtained in the band shift assay [26]. Thereafter, 1 μL of RNase A (1 mg/mL) was added to the nanocomplexes. Two siRNA-only controls were also established: a positive control, which was not subjected to RNase A treatment, and a negative control treated with RNase A. The reaction was allowed to proceed at 37 °C for 2 h and then terminated by the addition of EDTA (110 mM) to a final concentration of 10 mM (*v*/*v*). Sodium dodecyl sulphate (SDS, 6%) was then added to a final concentration of 0.5% (*v*/*v*). The reaction mixture was incubated at 55 °C for 20 min to permit the release of the siRNA from the nanocomplexes. Samples were then run on a 2% agarose gel and analysed as previously described (Section 2.4.1).

### 2.5. Cell Viability Studies

#### 2.5.1. Cytotoxicity Assay

Cells (2 × 10^4^ cells per well) were plated in 48-well plates containing 200 μL growth medium and incubated overnight to allow for attachment. FAuNCs (at the optimum, sub-optimum, and supra-optimum ratios) and Lipofectamine™ 3000 (LF3K) nanocomplexes (according to manufacturer’s instructions) were prepared. Untreated cells served as the control, which was assumed to have 100% cell survival. The assay was conducted in triplicate. Cells were incubated at 37 °C for 48 h, after which the spent medium was removed and replaced with fresh medium containing 20 μL MTT reagent (5 mg/mL in PBS). Cells were incubated at 37 °C for 4 h, after which the medium was removed and replaced with 200 μL DMSO. Plates were gently shaken to allow for the development of a clear, purple solution arising from the dissolution of the formazan crystals. The absorbance was read at 540 nm using a Mindray MR-96A microplate reader (Vacutec, Hamburg, Germany) against DMSO as a blank. Cell viability was calculated using Formula (3).

(3) $$Cell\;viability=\frac{A_{540}\;of\;treated\;cells}{A_{540}\;of\;control}\times 100\%$$

#### 2.5.2. Apoptosis Studies

Apoptosis was evaluated using the acridine orange/ethidium bromide (AO/EB) dual staining [27]. Cells were plated at a density of 2 × 10^5^ cells per well in a 24-well plate and incubated overnight. The medium was then replaced, and the cells were treated with nanocomplexes at the optimal ratios and incubated for 24 h. Subsequently, the medium was removed, and cells were washed with phosphate-buffered saline (PBS). Cells were stained with 15 μL of AO/EB dye (0.1 mg/mL: 0.1 mg/mL in PBS) for 5 min at room temperature, washed with PBS to remove unbound dye, and viewed using an inverted fluorescence microscope (CKX41, Olympus Corporation, Tokyo, Japan). Images were taken using Analysis Five Software 5.0 (Olympus Soft Imaging Solutions, Tokyo, Japan). The apoptotic index was calculated using Formula (4), where the total number of apoptotic cells included cells in both early and late apoptosis:

(4) $$\mathrm{Apoptotic}\;\mathrm{index}\;=\;\frac{\mathrm{number}\;\mathrm{of}\;\mathrm{apoptotic}\;\mathrm{cells}}{\mathrm{total}\;\mathrm{number}\;\mathrm{cells}\;\mathrm{counted}}$$

### 2.6. Cellular Uptake

Cells were plated and incubated as for the apoptosis assay. Thereafter, cells were treated with the FAuNCs complexed to 0.3 µg of BLOCK-iT™ Fluorescent Oligo and incubated for 24 h. Following incubation, the medium was removed, and cells were rinsed with PBS. Cells were viewed under a fluorescence microscope and images obtained as previously described (Section 2.5.2).

### 2.7. Flow Cytometry Studies

Flow cytometry is a technique that allows for the analysis of the characteristics of single cells in a population based on their fluorescence profiles [28]. All flow cytometry experiments were performed using the Guava Muse Cell Analyser (Luminex Corporation, Austin, TX, USA). Cells were plated in 24-well plates at a density of approximately 4 × 10^4^ cells per well and incubated overnight. Thereafter, cells were treated with TNF-α-targeted siRNA complexed with LF3K and FAuNC at the optimal ratios, and incubated for 48 h. The medium was then removed from the wells, and cells were washed with PBS. Cells were removed from the plate through trypsinisation, centrifuged at 300× *g* for 5 min, resuspended and treated according to the manufacturer’s protocols for the respective assays.

#### 2.7.1. Caspase 3/7 Analysis

Cells were resuspended in 50 μL of 1× Assay Buffer BA and treated with 5 μL of Muse^®^ Caspase-3/7 Reagent working solution. The samples were briefly vortexed and incubated at 37 °C for 30 min. Samples were then treated with 150 μL of Muse^®^ Caspase 7-AAD working solution, mixed, and incubated in the dark at room temperature for an additional 5 min. Analysis was then carried out using the Muse^®^ cell analyzer (Luminex, Austin, TX, USA).

#### 2.7.2. Cell Cycle Analysis

Trypsinized cells were washed with PBS and fixed in 70% ethanol at −20 °C overnight. Following cell fixation, the ethanol was removed by centrifugation at 300× *g* for 5 min, and the cells were washed with PBS. Cells were resuspended in 200 μL of the Muse^®^ Cell Cycle Reagent and incubated in the dark at room temperature for 30 min. Samples were analysed using the Muse^®^ cell analyzer.

#### 2.7.3. Oxidative Stress Analysis

After trypsinisation, the cells were resuspended in 1× Assay Buffer to a concentration of 1 × 10^6^ cells/mL. Approximately 10 μL of the cell suspension was then treated with 190 μL of the Muse^®^ Oxidative Stress Reagent working solution, mixed, and incubated at 37 °C for 30 min. Samples were analysed on the Muse^®^ cell analyzer.

### 2.8. Statistical Analysis

All assays were performed in triplicate, except for the gene expression assays, which were performed in duplicate. Data are presented as means ± SD. Statistical analysis was conducted using GraphPad Prism version 6.01 (GraphPad Software Inc., San Diego, CA, USA). Groups were compared using two-way analysis of variance (ANOVA) followed by Tukey’s multiple comparisons test. The * *p*-values < 0.05 and ** *p*-values < 0.01 were considered statistically significant.

## 3. Results and Discussion

### 3.1. Characterisation

#### 3.1.1. Optical Characterisation

**Figure 1:**
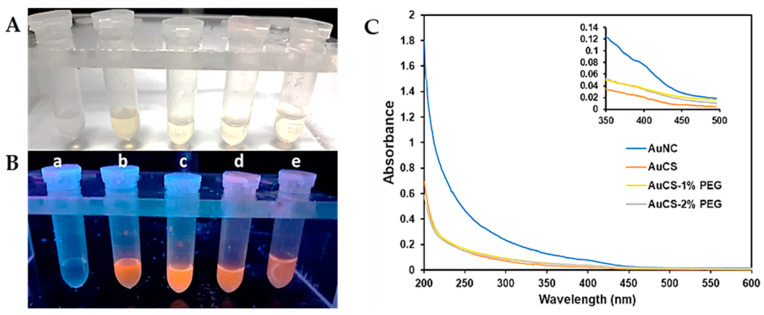
Comparison of the AuNC and FAuNC solutions under (**A**) visible light and (**B**) UV light (λ = 366 nm). For 1A and B-(a) H_2_O (b) AuNC (c) AuCS (d) AuCS-1% PEG (e) AuCS-2% PEG; (**C**) UV-vis spectra of AuNC and FAuNC. The inset shows a shoulder peak at approximately 390 nm in the AuNC spectrum (axes on the inset graph represent the same parameters as those on the main graph).

The synthesis of the AuNCs was confirmed visually by the appearance of a light-yellow solution after 24 h incubation at 70 °C. Upon irradiation with UV light (wavelength of 366 nm), the solutions emitted a bright orange fluorescence (Figure 1B). The AuCS fluoresced brighter than the AuNCs, which may be attributed to the phenomenon of restricted intramolecular motion. In the AuCS, the fluorescent AuNCs are confined within the CS polymer matrix by electrostatic bonds. This restricts the movement of capping ligands and prevents excited electrons from returning to the ground state by non-radioactive decay. This releases less energy than radioactive decay, thus enhancing fluorescence [25,29]. However, the fluorescence intensity of PEGylated AuNCs was weaker, suggesting that functionalisation with the CS-PEG disrupted the nanogel structures of the AuCS that amplified fluorescence.

The UV spectrum for the AuNCs showed an increase in absorbance from 450 nm but displayed no prominent peaks (Figure 1C). The absence of a localised surface plasmon resonance (LSPR) peak at approximately 520 nm and the onset of absorbance from approximately 450 nm further indicate that ultrasmall AuNCs, which absorb strongly in the UV range of the light spectrum, were synthesised [30]. A small shoulder peak, shown in the inset, can be observed at approximately 390 nm. Shoulder peaks at ~400 nm have been commonly reported in the UV-vis spectra of thiolate-capped AuNC [24,31,32,33,34]; and may be attributed to electron transfer between the ligand and metal or within the ligand, or due to electron excitation [35,36]. The absence of this shoulder peak in the spectra of the FAuNC may result from differences in charge transfer in the CS-coated AuNC.

#### 3.1.2. FTIR

**Figure 2:**
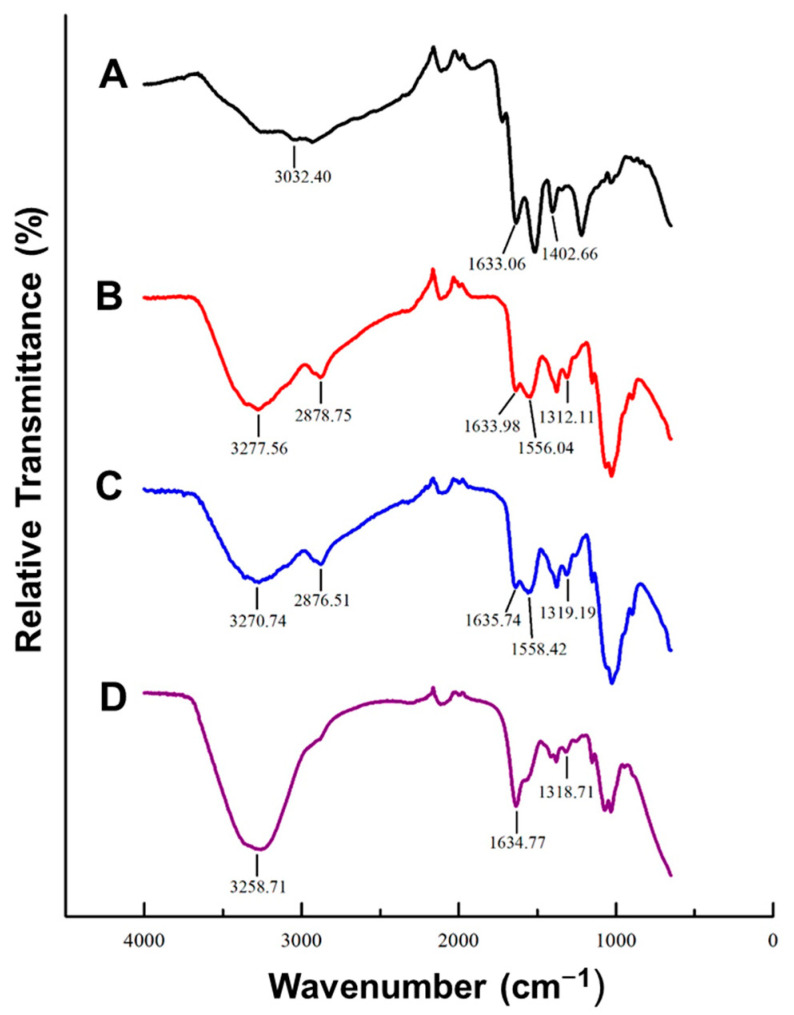
FTIR spectra of AuNC and FAuNC with major peaks labelled. (A) AuNC, (B) AuCS, (C) AuCS-1% PEG, and (D) AuCS-2% PEG.

FTIR provides information on the types of bonds present in NP, depending on their absorption of infrared radiation [37]. This allows for confirmation of functionalization. The spectra for the plain AuNC and FAuNC are shown in Figure 2. The successful synthesis of the AuNC by GSH reduction can be inferred from the presence of amide bonds indicated by the peaks at 1633.06 and 1402.66 cm^−1^, which correspond to C = O and C–N stretching, respectively [38]. The FTIR spectrum of plain GSH additionally displays a peak in the 2600–2550 cm^−1^ region corresponding to S-H stretching in the thiol group [38,39,40]. This peak cannot be observed in the spectrum of the AuNCs, indicating that the sulphur successfully bound the gold to form GSH-capped AuNCs.

The successful CS functionalisation is suggested by the change in the shape of the peak at 3277.56 cm^−1^ and the appearance of a small peak at 2878.75 cm^−1^ in the AuCS spectrum. These peaks are characteristic of polysaccharides and are present in the spectrum of pure CS, where they correspond to free amine and hydroxyl groups, and C-H stretching, respectively [41,42]. Their presence in the AuCS spectrum is thus indicative of successful attachment of the CS to the AuNC. The peaks at 1633.98 cm^−1^, 1556.04 cm^−1^, and 1312.11 cm^−1^ are also characteristic of CS, and indicate the C = O stretching of amide I, N-H bending in amide II, and C-N stretching in amide III, respectively [42,43].

PEG has been reported to display characteristic peaks at approximately 3430 cm^−1^, 2870 cm^−1^, and 1110 cm^−1^. These correspond to intra-molecular hydrogen bonds, C-H stretching, and C-O-C stretching within the PEG backbone, respectively [41,44,45]. However, these cannot be distinguished on the spectra for AuCS-1% PEG or AuCS-2% PEG, possibly due to masking by the CS.

#### 3.1.3. Sizing and Zeta Potential

**Figure 3:**
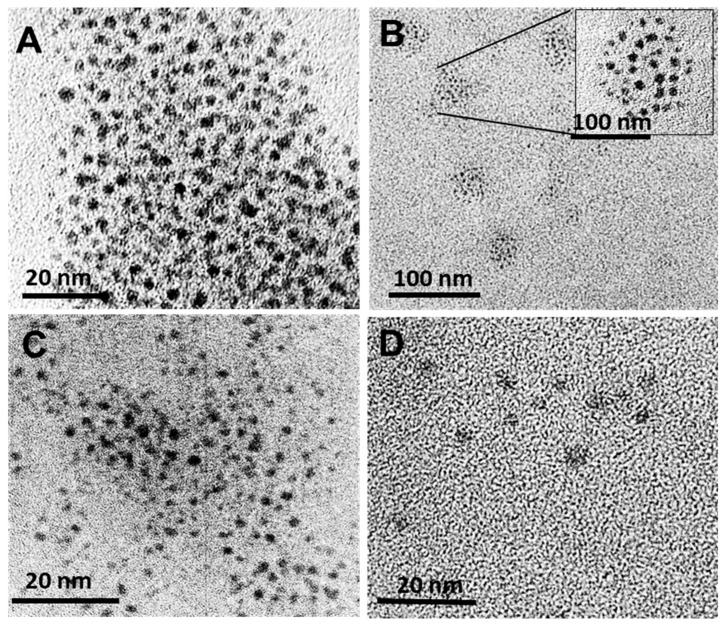
TEM images of AuNC and FAuNC. (**A**) AuNC, (**B**) AuCS, inset shows organisation of AuNC in circular AuCS nanogels, (**C**) AuCS-1% PEG, and (**D**) AuCS-2% PEG.

The TEM images for the plain AuNCs and FAuNCs are shown in Figure 3, and the sizes obtained from the TEM images are provided in Table 2. The AuNCs were circular and monodisperse. Their TEM size (1.86 nm) is close to the critical size at which energy levels become discrete, and is characteristic of AuNCs [46].

The size of the AuNCs did not change significantly following functionalisation. This is in accordance with the UV-vis spectra, which showed the absence of an LSPR peak for the AuNC and all FAuNC. The AuCS were observed to self-assemble into larger, spherical nanogels of approximately 21 nm in diameter, consisting of multiple AuNCs encapsulated within the CS polymer (Figure 3B). Several studies have reported the self-assembly of GSH-capped nanoclusters into nanogels with cationic polymers [25,47,48,49]. The confinement of AuNCs within these nanogels produces the amplified fluorescence observed in Figure 1B. PEGylation of the CS disrupted the formation of these nanogels, as shown in Figure 3C,D, correlating with the lowered fluorescence of the PEGylated AuNCs. Functionalization with the CS-PEG may have led to the formation of nanogels in which the AuNCs were less confined compared to the AuCS, allowing for energy release through non-radioactive decay.

Uncoated AuNCs had a hydrodynamic diameter of 73.6 nm and a zeta potential of −19.5 mV, due to the presence of anionic GSH peptides capping the AuNCs. Functionalization with CS led to an increase in the hydrodynamic diameter and zeta potential, indicating that the anionic AuNCs were successfully encapsulated within the CS polymer to form cationic nanogels. PEGylated FAuNCs displayed lower zeta potentials than AuCS, confirming the addition of the PEG chains to the amine groups of CS. The hydrodynamic diameters also decreased in a grafting density-dependent manner, to 194.5 nm for AuCS-1% PEG and 97.5 nm for AuCS-2% PEG. These size changes are consistent with reports on the swelling behaviour of nanogels. In aqueous solutions, CS hydro- and nanogels become hydrated and swell as water molecules move into the nanogel and interact with free hydrophilic amine groups [50,51]. As such, the hydrodynamic diameter of nanogels is often reported to be significantly larger than the TEM size [25,52,53,54]. This swelling behaviour is influenced by factors such as crosslinking and charge density [55]. Reduced swelling capacity has thus also been observed for increasing grafting densities of PEG, consistent with the size changes reported for PEGylated FAuNCs [56].

The zeta potentials of all nanocomplexes dropped to negative or close to neutral, indicating successful complexation with siRNA. As with the uncomplexed FAuNC, PEGylation shielded the surface charge, resulting in increased, near-neutral zeta potentials compared to the AuCS:siRNA. The AuCS and AuCS-1% PEG formed highly condensed nanocomplexes, as suggested by the decrease in hydrodynamic diameter following complexation. The AuCS decreased from 267.8 nm to 97.7 nm, and the AuCS-1% PEG decreased from 194.5 nm to 108.5 nm. In contrast, the AuCS-2% PEG increased in size following complexation. This may be due to its smaller size (97.5 nm) and limited swelling capacity in solution compared to the other FAuNCs, which may have limited its condensation ability.

### 3.2. Binding Studies

#### 3.2.1. Band Shift Assay

**Figure 4:**
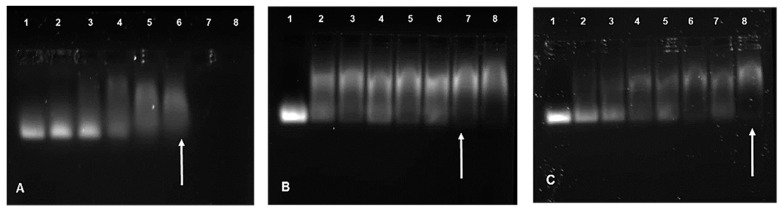
Band shift assay of (**A**) AuCS, (**B**) AuCS-1% PEG, and (**C**) AuCS-2% PEG run on a 2% agarose gel. Lane 1 contains uncomplexed siRNA, lanes 2–8 contain increasing siRNA:FAuNC weight ratios. Endpoint ratios are indicated by arrows.

All FauNCs were able to successfully bind siRNA (Figure 4). The AuCS exhibited complete binding at a relatively low siRNA:FAuNC ratio of 1:2.5, due to its high charge density and zeta potential, which promoted interactions with the siRNA. The high swelling capacity suggested by NTA sizing may have further allowed the siRNA to move and bind within the nanogels. The addition of PEG to the AuCS increased the binding ratios to 1:10.5 and 1:8.5 for AuCS-1% PEG and AuCS-2% PEG, respectively, corresponding to the reduced zeta potentials observed for the PEGylated FAuNC. The mechanisms by which PEGylation reduces zeta potential also inhibit interactions with nucleic acids, and PEGylation of cationic polymers has thus often been reported to reduce their nucleic acid binding abilities [57,58,59]. PEG has been known to mask some of the amine groups of cationic polymers, thereby affecting the optimal binding ratio [60].

#### 3.2.2. EB Intercalation Assay

**Figure 5:**
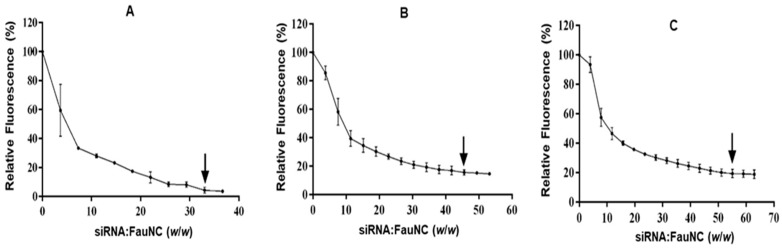
The EB intercalation assays for (**A**) AuCS, (**B**) AuCS-1% PEG, and (**C**) AuCS-2% PEG. Points of inflection are indicated by arrows.

Efficient nucleic acid condensation facilitates the formation of small nanocomplexes in which the payload is protected from degradation. However, efficient siRNA condensation is hampered by its short, rigid structure and low charge density [61]. Moreover, siRNA that is condensed too tightly by the carrier may be prevented from being released into the cytoplasm after cellular uptake, thus reducing transfection efficiency [62]. The EB intercalation assay was thus conducted to assess the abilities of the FAuNCs to condense siRNA (Figure 5).

All FAuNCs were able to condense the siRNA to a significant degree, with fluorescence decays of 80% and above (Table 3). The AuCS displayed the strongest condensation ability, condensing the siRNA at the lowest siRNA:FAuNC ratio (1:33) and to the greatest degree (95.6% dye displaced). This was expected due to their strong positive charge, which enhanced siRNA condensation and nanogel structure, allowing the formation of compact nanocomplexes. PEGylation inhibited the ability of the FAuNCs to condense siRNA in a grafting density-dependent manner, with PEGylated FAuNCs showing lower fluorescence decays than the AuCS nanocomplexes, possibly due to their reduced charge density.

#### 3.2.3. Nuclease Protection Assay

**Figure 6:**
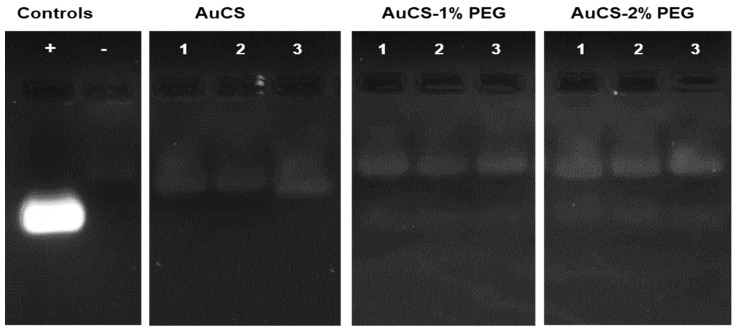
RNase protection of siRNA by the FAuNC nanocomplexes. Positive (+) control = untreated siRNA; negative (−) control = RNase-treated siRNA; lanes 1, 2, and 3 contain the sub-optimal, optimal, and supra-optimal ratios, respectively, of the indicated FAuNC.

The nuclease protection assay assessed the ability of the FAuNCs to protect the siRNA payload from degradation by RNase A. The AuCS were able to provide protection from complete degradation without the need for stabilizers (Figure 6). This may have been due to the incorporation of the siRNA into the nanogel, where it was less exposed to RNases. The FAuNC-protected siRNA may also have assumed a conformation that prevented RNase binding [63], such as the stiffening of the siRNA, which prevents its binding to the active site of the enzyme. Protection by the PEGylated FAuNCs may be due to a combination of compaction by CS and stabilisation by PEG [64].

PEGylation did not show improved protection (Figure 6). The bands for the PEGylated FAuNC have the same fluorescent intensity as those for the AuCS. This suggests that both unPEGylated and PEGylated FAuNCs provided a similar degree of protection against RNase degradation. However, it is possible that the effects of PEGylation may be more apparent when tested in an in vivo setting, where the PEG chains may provide greater protection against degradation by preventing nanocomplex destabilisation in the plasma.

### 3.3. Cytotoxicity Studies

#### 3.3.1. MTT Cytotoxicity Assay

**Figure 7:**
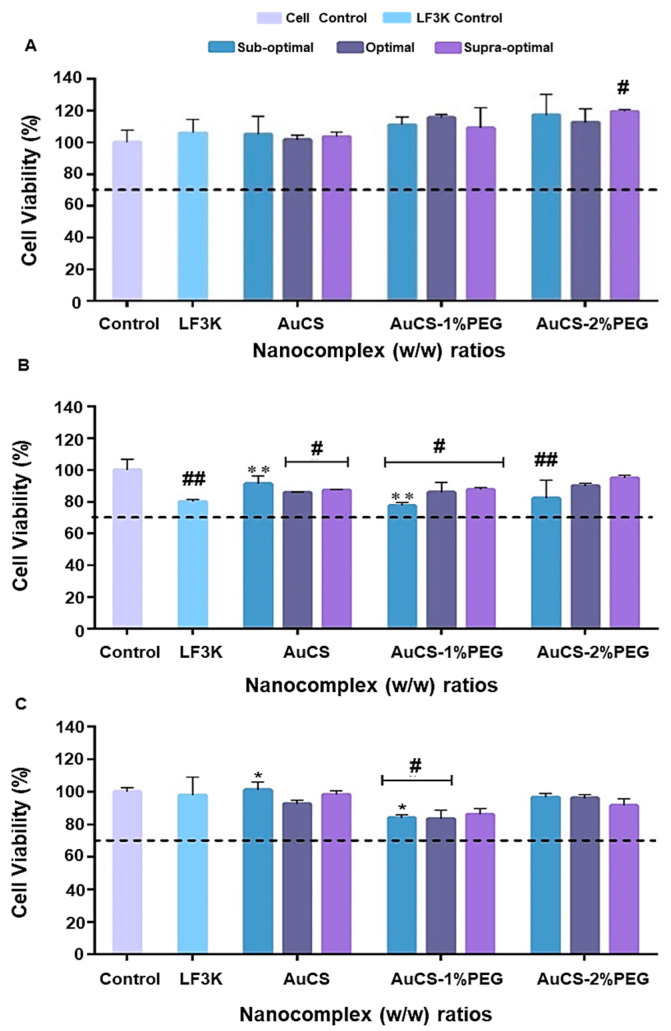
Cell viability assay in: (**A**) HEK293, (**B**) MCF-7, and (**C**) MDA-MB-231 cells. Control=cells only, LF3K = Liofectamine control, (---) signifies 70% cell viability. Data are represented as mean ± SD (*n* = 3) * *p* < 0.05, ** *p* < 0.01 considered statistically significant between nanocomplex ratios; # *p* < 0.05, ## *p* < 0.01 considered statistically significant vs. the control.

Nanocarriers for siRNA delivery should ideally be non-toxic, so that any effects observed after transfection can be attributed to the effects of gene knockdown. The toxicity of the FAuNCs was assessed using the MTT assay in the HEK293, MCF-7, and MDA-MB-231 cells. According to the International Standards Organisation, medical devices that reduce cell viability by 30% or more can be considered cytotoxic [65]. From Figure 7, it can be seen that cell viabilities remained above this threshold, indicating that all nanocomplexes were non-toxic in all cells. These results were expected given the biodegradability and biocompatibility of the individual components of the FAuNCs. While highly cationic polymers may induce toxicity by disrupting the cell membrane, the reduced charge density of the CS following PEGylation and siRNA complexation might have attenuated this toxicity [66].

The non-toxicity of the AuCS is consistent with previous reports on CS-functionalised AuNCs, with cell viabilities of over 90% reported in breast cancer (MCF-7), cervical cancer (HeLa), and mouse embryonic fibroblast (3T3) cells [67]. Furthermore, AuNCs@CS-TPP have also been reported to be non-toxic to HeLa and liver cancer (HepG2) cells [68]. CS on its own was reported to produce little or no toxicity when tested in vitro in HEK293, HeLa, and MCF-7 cells, but was least tolerated in the HepG2 cells [69]. The biocompatibility and biodegradability of CS have influenced its biomimetic use in wound healing applications, commonly in hydrogel, nanogel or cryogel preparations [50,70,71].

Cell-line-specific interactions were observed after treatment with the PEGylated FAuNCs. PEGylation enhanced cell viability in HEK293 cells, whereas the AuCS-1% PEG nanocomplexes resulted in slightly reduced viability in cancer cells. This may have been due to the higher amount of AuCS-1% PEG required to fully bind the siRNA, which may have slightly exacerbated any toxicity due to CS or PEG. The 2% PEG coating attenuated the toxicity levels induced by the AuCS-1% PEG, possibly by greater shielding of the FAuNC surface and inhibiting cellular interactions to a greater extent. The overall biocompatibility of the PEGylated AuNCs is nevertheless notable given that more of the carrier was required to fully bind the siRNA.

#### 3.3.2. Apoptosis Studies

**Figure 8:**
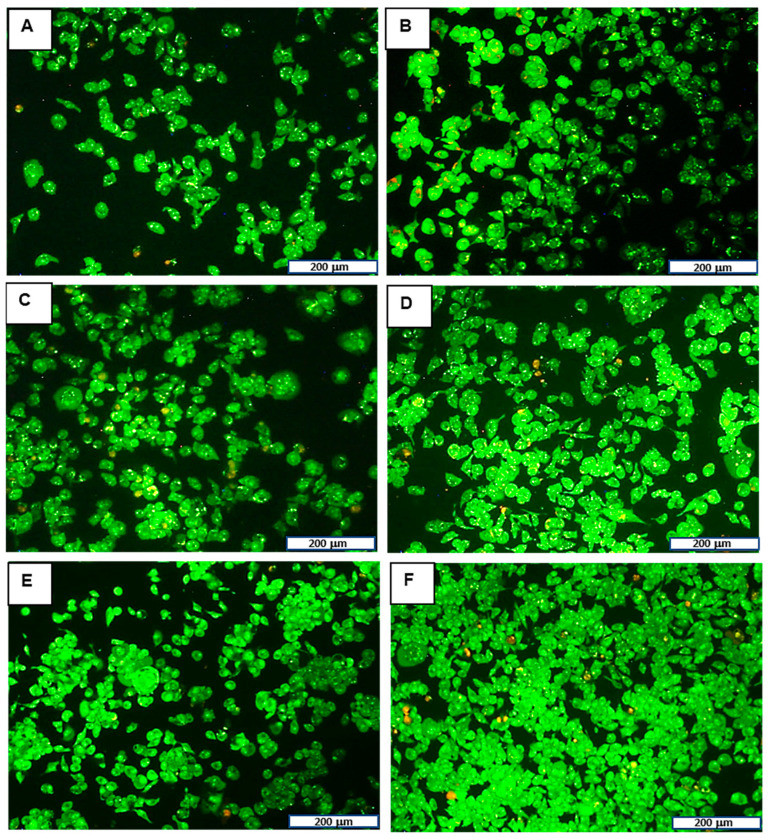
Dual EB/AO assay in MDA-MB-231 treated with (**B**) free siRNA, (**C**) LF3K, (**D**) AuCS, (**E**) AuCS-1% PEG, and (**F**) AuCS-2% PEG complexes. (**A**) represents the untreated control. The scale bar represents 200 μm for all images.

Dual AO/EB staining was performed to determine whether any reductions in cell viability observed in the MTT cytotoxicity assay were due to the induction of apoptosis in response to FAuNC treatment or necrosis. Apoptosis is a form of programmed cell death that eliminates damaged or abnormal cells [72]. Necrosis is a non-specific form of cell death and is generally undesirable, as it may promote inflammation [73]. The assay was conducted using the optimum ratios in the MDA-MB-231 cells, as they are the TNF-α-producing TNBC cells. This allowed for confirmation of the non-toxicity of the FAuNCs before investigation of the effects of anti-*TNF-α* siRNA delivery. The results are shown in Figure 8, and the apoptotic indices are presented in Table 4.

The results show that the FAuNCs were non-toxic at the ratio tested. Treatment with LF3K and FAuNC nanocomplexes led to small increases in apoptosis levels. However, levels remained below 5% and were comparable to those of the untreated control cells. Cells treated with AuCS-2% PEG exhibited more apoptosis, yet remained non-toxic and comparable to the LF3K. This correlates with the results from the cytotoxicity study.

### 3.4. Cellular Uptake

**Figure 9:**
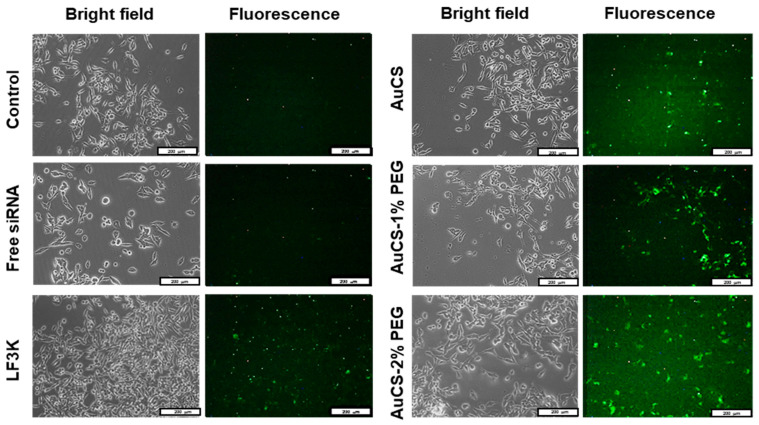
Cellular uptake of FAuNC and LF3K complexed with BLOCK-iT™ fluorescent siRNA in HEK293 cells. Images were taken on a fluorescence microscope. The scale bar represents 200 μm for all images.

**Figure 10:**
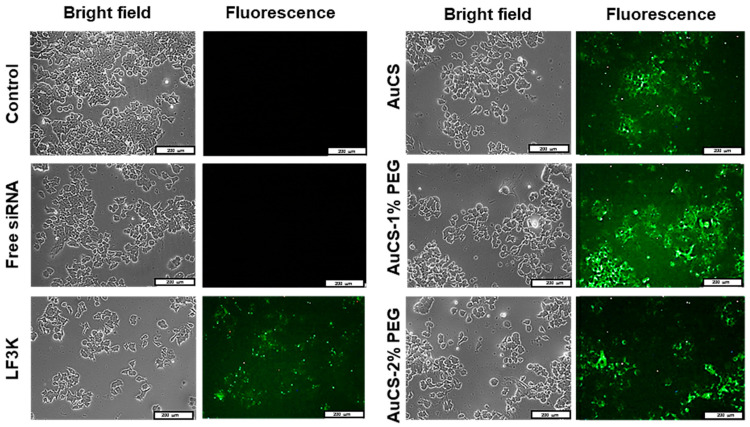
Cellular uptake of FAuNC and LF3K complexed with BLOCK-iT™ fluorescent siRNA in MCF-7 cells. Images were taken on a fluorescence microscope. The scale bar represents 200 μm for all images.

**Figure 11:**
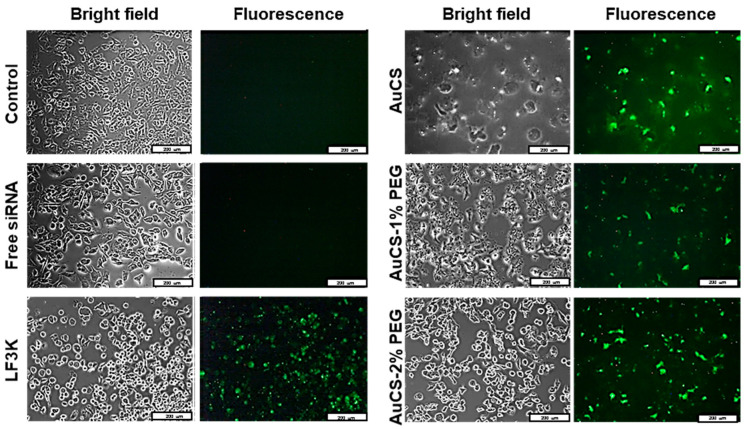
Cellular uptake of FAuNC and LF3K complexed with BLOCK-iT™ fluorescent siRNA in MDA-MB-231 cells. Images were taken on a fluorescence microscope. The scale bar represents 200 μm for all images.

Cellular uptake of FAuNC:siRNA nanocomplexes was assessed qualitatively using a siRNA labelled with a fluorescent tag. The images obtained for the HEK293, MCF-7, and MDA-MB-231 cells are shown in Figure 9,Figure 10 and Figure 11, respectively. The presence of fluorescence indicated that all FAuNCs were able to effectively interact with cells. The level of fluorescence was comparable to that of LF3K-treated cells. In contrast, untreated cells and cells treated with naked siRNA show no fluorescence.

The choice of uptake pathway is influenced by the characteristics of the nanocomplexes, such as size, composition, and rigidity, although conflicting results have been reported regarding the influence of these factors [74,75,76]. While it is likely that the nanocomplexes primarily exploit passive pathways, it is also possible that receptor-mediated endocytosis may also be utilised to some extent due to the adsorption of serum proteins [77]. The uptake of the nanocomplexes is notable given their weakly negative and neutral zeta potentials, which may have impaired interactions with the cell membrane. However, it is possible that the zeta potentials of the nanocomplexes may have differed in the cell growth medium or in vivo environments due to the formation of an electrical double layer as ions interact with the NP surface, and the adsorption of serum proteins onto the NP surface [78]. Differences in zeta potentials for gold and metal oxide NPs dispersed in water and in both serum-free and supplemented medium have been widely reported [78,79,80,81].

### 3.5. Flow Cytometry Studies

#### 3.5.1. Caspase 3/7 Activity

**Figure 12:**
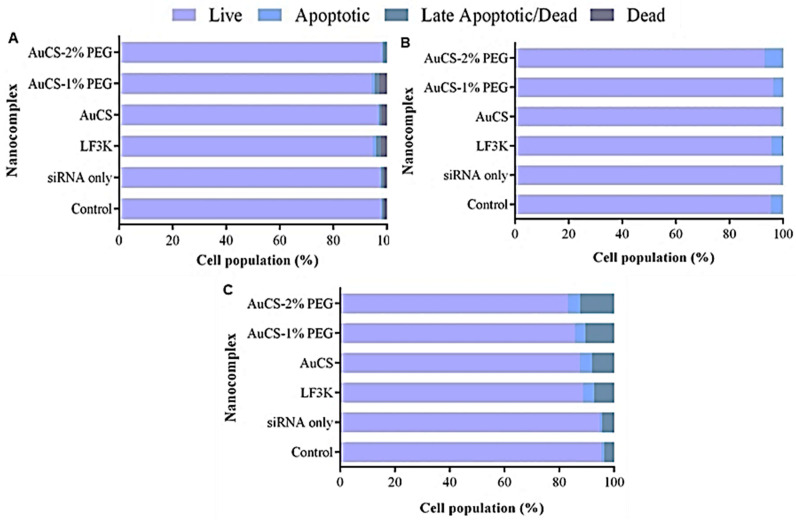
Graphical representation of the apoptotic profiles following *TNF-α* knockdown in (**A**) HEK293, (**B**) MCF-7, and (**C**) MDA-MB-231 cells. Control = cell-only control; siRNA only = anti-*TNF-α* siRNA only; LF3K = Lipofectamine 3000.

The activation of effector caspases 3 and 7, which are involved in the caspase cascade that initiates apoptosis, in response to *TNF-α* knockdown was evaluated using the Muse^®^ Caspase-3/7 assay kit. FAuNC-mediated delivery of the anti-*TNF-α* siRNA was found to be non-toxic to the HEK293 cells. As shown in Figure 12, over 94% of the cell population remained viable after treatment.

Caspase 3/7 activation was similarly low in the MCF-7 cells, with the proportion of live cells in all samples similar to or exceeding that of the control. In contrast, anti-*TNF-α* siRNA delivery in the MDA-MB-231 cells was observed to lead to slightly increased levels of Caspase 3/7 activation compared to the control. AuCS and LF3K-mediated delivery produced similar increases in caspase 3/7 activation, resulting in a rise in the percentage of apoptotic cells from 4.4% in the control to 12.4% and 11.3%, respectively. The PEGylated FAuNC produced slightly higher apoptosis levels of 14.1% and 16.7% for AuCS-1% PEG and AuCS-2% PEG, respectively.

These results are consistent with previous reports of apoptosis induction following TNF-α inhibition through the use of inhibitors [82], antibody targeting [83], and gene knockdown [84]. Analysis of gene expression following knockdown showed downregulation of TRADD, indicating inhibition of TNF-α signalling, and upregulation of p53 and several genes involved in apoptosis, including TNF-related apoptosis-inducing ligand (*TRAIL*) [84]. TRAIL selectively induces apoptosis in cancer cells through the receptors TRAIL-R1 and TRAIL-R2 [85]. TNF-α knockdown thus led to reduced activation of the cell growth and inflammatory pathways transcribed by NF-κB, which, together with the increased expression of TRAIL, resulted in the activation of apoptotic pathways.

#### 3.5.2. Cell Cycle Analysis

**Figure 13:**
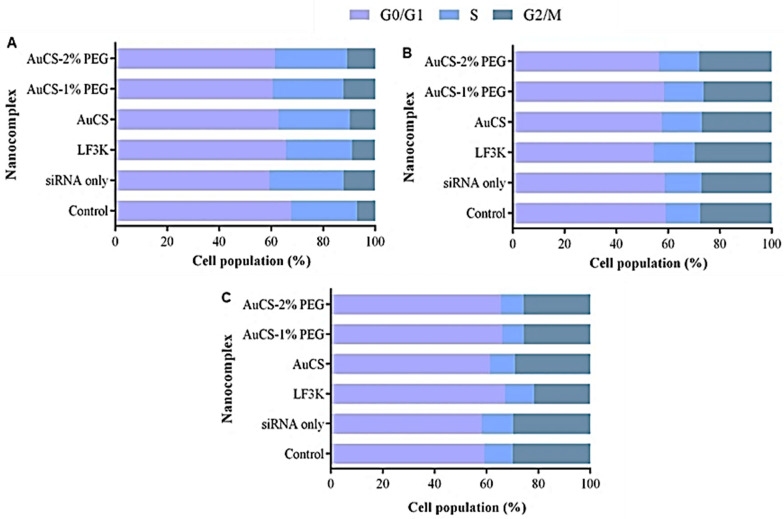
Graphical representation of the cell cycle distribution following TNF-α knockdown in (**A**) HEK293, (**B**) MCF-7, and (**C**) MDA-MB-231 cells. Control = cell-only control; siRNA only = anti-TNF-α siRNA only; LF3K = Lipofectamine 3000.

The HEK293 and MCF-7 cells were minimally affected by treatment with the *TNF-α*-targeting FAuNC nanocomplexes (Figure 13). The HEK293 cells displayed a slight increase in the proportion of cells in the S and G2 phases compared to the control, suggesting that the cells were actively proliferating and were not adversely affected by the nanocomplexes. In contrast, delivery of anti-*TNF-α* siRNA to MDA-MB-231 cells resulted in a slight increase in the proportion of cells in the G0/G1 phase, accompanied by a slight decrease in cells in the S and G2/M phases. This shift was minimal for the AuCS-treated cells, which showed an increase from 59.2% cells in the G0/G1 phase for the control to 61.3%. The shift was slightly more prominent for cells treated with the PEGylated FAuNCs, producing shifts equivalent to those observed for the LF3K-mediated knockdown. The observed proportion of cells in the G0/G1 phase following treatment was 67.1% for LF3K, 66.1% for AuCS-1% PEG, and 65.5% for AuCS-2% PEG.

**Figure 14:**
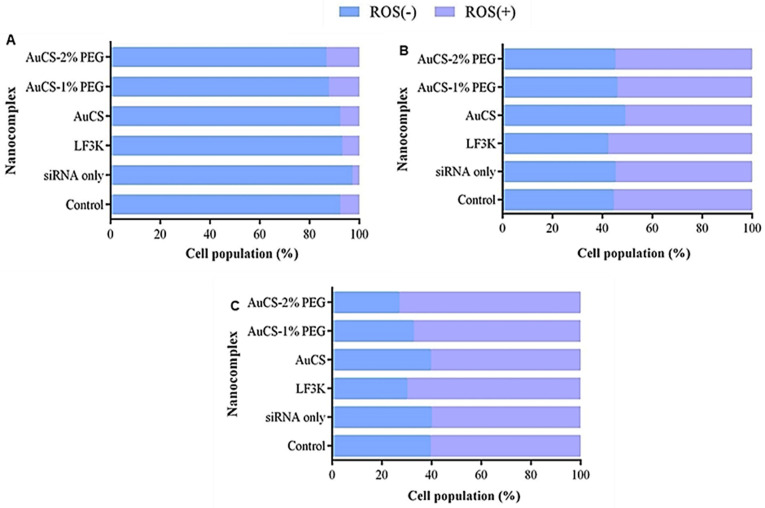
Graphical representation of the ROS levels following TNF-α knockdown in (**A**) HEK293, (**B**) MCF-7, and (**C**) MDA-MB-231 cells. Control = cell-only control; siRNA only = anti-TNF-α siRNA only; LF3K = Lipofectamine 3000.

A shift in the distribution of the cell population in the different cell cycle phases may occur if a treatment alters the expression of genes involved in cell cycle progression. NF-κB has been implicated in the regulation of several proteins involved in cell cycle progression, including the cyclin D proteins and c-MYC oncogene [86,87]. TNF-α may thus promote cell cycle progression by activating the NF-κB pathway. TNF-α treatment in MDA-MB-231 and MCF-7 cells, for example, has been reported to shift cells from the G0 to the S phase, sensitising them to chemotherapy [88]. Several studies have also reported cell cycle arrest following the knockdown of TNF-α. It was observed that TNF-α-knockout leukaemia cells displayed a shift to the G0 phase compared to wild-type cells. This was accompanied by a decrease in the expression of CDC6 and CDC23 proteins, which are involved in mediating cell cycle progression. Knockout was thus suggested to lead to cell cycle arrest [89]. A similar shift was observed in the MDA-MB-231 cells, where shRNA-mediated knockdown of tmTNF-α resulted in an increase in cells in the quiescent G0 phase and a decrease in cells in the S phase [90]. These results are similar to those shown in the ensuing assay (Figure 14C) and suggest that PEGylated FAuNC-mediated delivery of the anti-TNF-α siRNA selectively induced cell cycle arrest in the MDA-MB-231 cells.

#### 3.5.3. Oxidative Stress

Oxidative stress is a crucial process which leads to serious diseases and imbalances in the body [18]. Most of the HEK293 cells displayed reduced oxidative stress (Figure 14). The percentage of the cell population with elevated ROS remained below 10% for all samples except for the PEGylated FAuNC-treated samples, which showed slightly increased ROS(+) populations of 12.1–13.1% compared to the control (7.6%). There was little change in the distribution of the ROS(+) and ROS(−) populations in the luminal MCF-7 cells (Figure 14B). Treatment of MDA-MB-231 cells with the anti-*TNF-α* siRNA:FAuNC nanocomplex resulted in an increase in the proportion of the cell population with elevated ROS levels, from 60.8% in the control to 60.7%, 67.5%, and 73.4% for the AuCS, AuCS-1% PEG, and AuCS-2% PEG samples, respectively. The levels produced by the PEGylated FAuNC were similar to those of LF3K, where 70.2% of cells were ROS(+).

TNF-α induces intracellular ROS generation and antioxidant expression through NF-κB [91]. TNF-α-induced ROS generation is implicated in the pathology of inflammatory and cardiovascular disorders. Studies using in vitro and in vivo models have shown that TNF-α inhibition reduces oxidative stress [92,93,94]. Increased ROS generation induced by TNF-α stimulation in liver cancer cells has been observed to lead to increased cell migration [95]. It was thus unexpected to observe an increase in the ROS (+) population in MDA-MB-231 cells in response to anti-*TNF-α* siRNA delivery. This could indicate that the delivery of anti-*TNF-α* siRNA might lead to increased TNBC cell proliferation and potentially increased TNF-α release. The increased ROS in MDA-MB-231 cells, however, correlated with the results of the other flow cytometry assays, which indicated a decrease in cell proliferation. Cancer cells must still maintain ROS homeostasis to prevent the excessive levels that induce cell death, and tumours are still sensitive to exogenous treatments that induce ROS [96]. Studies have linked treatment-induced ROS generation in MDA-MB-231 cells to apoptosis [97,98]. Elevated ROS levels may also trigger apoptosis by damaging cellular macromolecules or disrupting the mitochondrial membrane [99].

It is essential to acknowledge the dual nature of ROS therapy. Treatments that induce only moderate increases in ROS levels may promote malignancy rather than apoptosis. Importantly, the HEK293 cells showed no increase or only a slight increase in ROS levels following treatment. This would suggest that the FAuNC are non-toxic and may not significantly impact expression pathways in non-cancer cells. Overall, FAuNC were consistently well-tolerated in the non-cancer HEK293 cells, indicating their safety. The effects of the anti-*TNF-α* appeared to be selective to the TNBC MDA-MB-231 cells. Studies have reported low or absent TNF-α production in HEK293 and MCF-7 cells [100,101]. This may suggest the success of the vector in delivering anti-*TNF-α* siRNA and initiating gene silencing. However, the shifts were minimal, suggesting that the knockdown needs to be improved.

## 4. Conclusions

Ultra-small AuNCs were successfully synthesised and functionalised with CS and PEG. The FAuNCs showed strong fluorescence upon excitation with UV light and were able to bind and condense siRNAs into compact nanocomplexes with ideal sizes for cellular uptake. In vitro toxicity studies demonstrated that FAuNCs were well-tolerated in all cells tested, eliciting no significant cytotoxicity or apoptosis induction, and were capable of interacting with cells to facilitate uptake. These characteristics indicate their suitability as delivery vehicles for therapeutic nucleic acids and their potential as live cell imaging agents. Preliminary knockdown studies using flow cytometry to evaluate the effects of anti-TNF-α siRNA delivery confirmed the safety of the FAuNCs in non-cancer cells and showed selective inhibition of MDA-MB-231 cell growth. Since these effects were minimal, further studies are required to optimise these FAuNCs for siRNA delivery. The composition of the FAuNCs can be optimised to improve the physical characteristics and protective abilities of the nanocomplexes. Quantitative assays and more thorough investigations into the uptake and intracellular trafficking pathways utilised by the FAuNCs may be conducted to accurately assess cellular uptake levels. Analysis of the release kinetics will also provide information on the rate of vector unpacking, as slow siRNA release may result in inefficient knockdown. Overall, all FAuNCs formulated and tested in this study were capable of interacting with siRNAs and showed favourable characteristics in vitro in breast cancer and non-cancer cells. However, further optimisation would be needed to take this system further as a delivery vector in gene silencing therapy.

## Figures and Tables

**Table 1:** Sequences of non-targeted and individual siRNA duplexes in the SMARTpool mixture.

siRNA Reagent	Sequence (5′–3′)
siGENOME non-targeted siRNA	UAGCGACUAAACACAUCAA
ON-TARGET plus Human TNF SMARTpool siRNA	GCCCGACUAUCUCGACUUU
	GCGUGGAGCUGAGAGAUAA
	UGACAAGCCUGUGCCCAU
	CCAGGGACCUGUGUGUAAU

**Table 2:** Sizes and zeta potentials of AuNCs and FAuNCs as determined by TEM and NTA (hydrodynamic size).

	Nanocluster			Nanocomplex	
	TEM Size (nm ± SD)	Hydrodynamic Size (nm ± SD)	Zeta Potential (mV ± SD)	Hydrodynamic Size (nm ± SD)	Zeta Potential (mV ± SD)
AuNC	1.86 ± 0.45	73.6 ± 4.7	−19.5 ± 7.5	-	-
AuCS	(single) 1.82 ± 0.37 (gel) 21.23 ± 3.83	267.8 ± 34.4	31.9 ± 0.1	97.7 ± 8.0	−8.0 ± 0.2
AuCS-1% PEG	1.89 ± 0.29	194.5 ± 24.7	13.9 ± 0.3	108.5 ± 16.2	−0.8 ± 0.9
AuCS-2% PEG	1.99 ± 0.38	97.5 ± 9.2	17.1 ± 2.2	151.5 ± 0.3	−1.6 ± 0.0

**Table 3:** The siRNA:FAuNC (*w*/*w*) ratios and decays in fluorescence observed at the point of inflection for all nanocomplexes in the EB intercalation assay.

Nanocomplex	siRNA:FAuNC (*w*/*w*) at Endpoint	Maximum Dye Displacement (% ± SD)
AuCS	1:33	95.6 ± 2.1
AuCS-1% PEG	1:45.6	84.4 ± 1.8
AuCS-2% PEG	1:55.1	80.6 ± 2.9

**Table 4:** Apoptotic indices in the MDA-MB-231 cells treated with the FAuNC nanocomplexes.

Treatment	Apoptotic Index
Control	0.03
Free siRNA	0.02
LF3K	0.04
AuCS	0.03
AuCS-1% PEG	0.02
AuCS-2% PEG	0.04

## Data Availability

The raw data supporting the conclusions of this article will be made available by the authors on request.

## Abbreviations

The following abbreviations are used in this manuscript:

AO	Acridine orange
AuNC	Gold nanoclusters
CS	Chitosan
EB	Ethidium bromide
FAuNC	Functionalised gold nanoclusters
FTIR	Fourier-transform infrared spectroscopy
NTA	Nanoparticle tracking analysis
PEG	Polyethylene glycol
RNAi	RNA interference
ROS	Reactive oxygen species
siRNA	Small interfering RNA
TEM	Transmission electron microscopy
TNBC	Triple-negative breast cancer
TNF-α	Tumour necrosis factor α
UV-Vis	Ultraviolet–visible spectroscopy
